# Defensive Glands in the Adult and Larval Stages of the Darkling Beetle, *Luprops tristis*

**DOI:** 10.1673/031.010.0701

**Published:** 2010-02-25

**Authors:** P. Abhitha, K.V Vinod, T.K. Sabu

**Affiliations:** Litter Entomology Research Unit, Post Graduate & Research Department of Zoology, St. Joseph's College, Devagiri, Calicut, Kerala, 673008, India.

**Keywords:** rubber plantations, litter beetle, Mupli, aggregation, dormancy

## Abstract

Invasion by large populations of the litter-dwelling darkling beetle *Luprops tristis* Fabricius (Coleoptera: Tenebrionidae) following the short spell of summer rains during April, and their extended state of dormancy is a regular event in rubber plantation habitats in south-western India. Strong smelling secretions of the beetle cause blisters on skin of human beings. Such secretions appear defensive because they appear to facilitate their avoidance by other predatory organisms. Defensive glands in the larvae and adults of *L. tristis* are described, as well as the mode of eversion of the glands. The glands in larvae consist of two pairs of noneversible glands in a conical depression on the 2^nd^ and 3^rd^ sternites, whereas in adults only one pair occurs between 7^th^ and 8^th^ sternal segments. These glands may be a major reason for avoidance of larvae and adults by their natural enemies and their very high numbers in the litter of rubber plantations.

## Introduction

Seasonal invasion by the litter dwelling darkling beetle, *Luprops tristis* Fabricius 1801 (Coleoptera: Tenebrionidae) (locally called ‘*mupli*’), of between 0.5 and 4 million/building immediately after summer rains is a regular event near and within rubber plantation tracts in the western slopes of South-Western Ghats. The continued presence of these nocturnally active beetles inside living areas is a nuisance. Although, these beetles neither sting nor bite, when disturbed by hand-picking them from walls or when they are squashed, they release an irritating, strong-smelling secretion that causes blisters to humans. Observations of *L. tristis* revealed neither any vertebrate nor invertebrate predators prevalent in rubber litter floor or in the buildings fed on *L. tristis* (personal observations). It has been suggested by Sabu *et al.* (2008) that this lack of predation could be a key reason for the massive population build up of *L. tristis* in rubber plantation belts. Moreover, the larvae, on being disturbed, release a strong smelling secretion indicating the presence of defensive glands that may deter predators. Defensive-compound secreting glands in Tenebrionidae are everted by haemolymph pressure (Tschinkel 1975) and the secretion is released involuntarily during stress when being predated upon (Kendall 1968, 1974). As these glands have not been described in larvae of *L. tristis*, the present study was undertaken to analyze the structure of defensive glands in adult and larval stages of *L. tristis*.

## Methods and Materials

A total of 20 freshly collected adult beetles that were sexed based on Vinod *et al.* (2008), and 20 fourth and fifth instar larvae, were killed using diethyl ether. Killed adults were pinned to a wax tray, the elytra and terga were removed exposing internal structures. Reproductive and digestive structures and fat reserves were removed to expose defensive glands. After washing in water followed by 70% alcohol, the sixth and seventh sternites with attached glands were separated from the rest of the sternites. The defensive glands were separated from the sterna by cutting along the posterior margin of the seventh sternum.

Killed larvae were pinned on a wax tray and were cut along the mid—dorsal side to expose the glands. The second and fourth sternites with attached glands were separated. Glands from both adults and larvae were dehydrated in graded series of ethyl alcohol, and brought to xylene through alcohol: xylene (1:1) mixture and they were mounted on glass slides in Canada balsam.

Discharge of gland secretions was observed by gently pressing the abdomen of live larvae and adults. The adult or larva was held between the left thumb and index finger, placed on the stage of a stereo zoom microscope (Labomed CZ 70; Labomed India Ltd, http://www.labomed.in) with the ventral surface of the insect facing up and keeping the posterior end away from the observer. When pressed, usually both larvae and adults discharged secretions and on pressing with modest pressure, glands were extruded in adults alone. Care was taken to immobilize the hind legs of adults with forceps, to avoid rupturing the gland reservoirs as the release of the oily secretion renders further observations difficult. Line drawings of the adult and larval gland were reconstructed from digital images.

## Results and discussion

**Figure 1:**
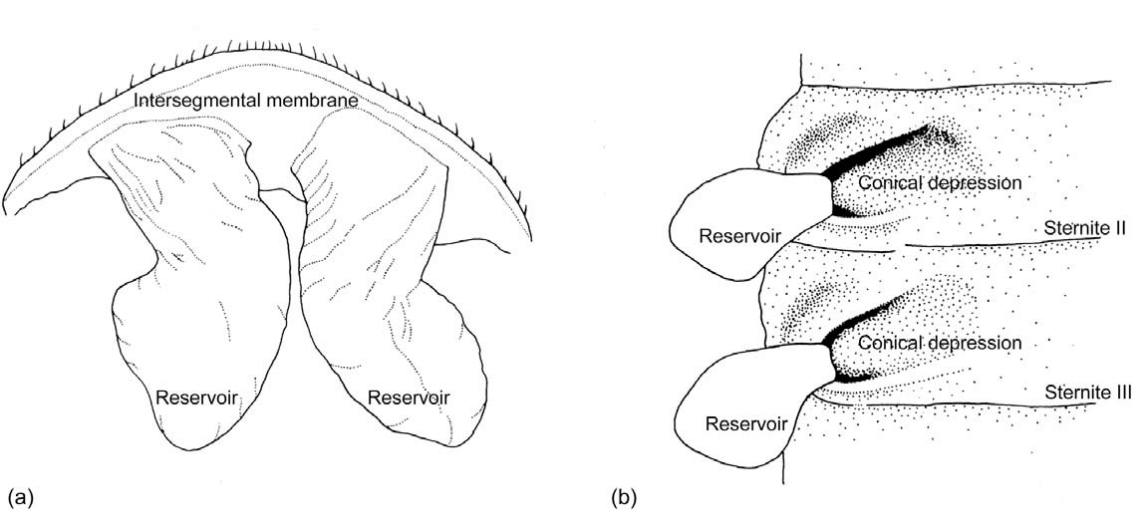
Line diagram of defensive gland of *Luprops tristis*. (a) Adult (dorsal view of the gland reservoirs cut from the remainder of the sternites); (b) Larva (view of paired glands of one side from the inside of the body and the reservoirs reflected to the exterior). High quality figures are available online.

**Figure 2:**
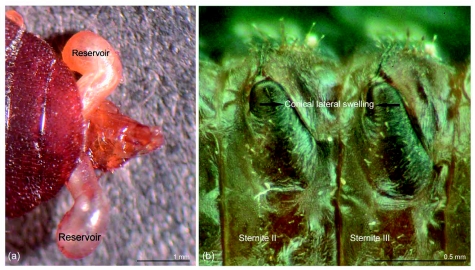
External view of defensive gland of *Luprops tristis*. (a) Adult; (b) lateral external swellings on sternite 2 and 3 of larva (paired swellings of one side). High quality figures are available online.

The structure of defensive glands is same in larvae and adults, but the location, alignment, number of glands and pattern of discharge of secretion vary. The gland in adults consists of a pair of small (0.8–0.9 mm), strongly wrinkled conical pouches that open independently; these glands occur parallel to the long axis of the body (Figure 1a, 2a). Glands in adults are evaginations of the intersegmental membrane between the seventh and eighth sternites and occur on either side of the hind gut immersed in a thick matrix of fat reserves. The secretion is produced by gland cells that cover the dorsal surface of the reservoirs. The opening of the glands is directed backwards and on pressing the abdomen the glands are everted (Figure 2a). In addition, when strongly disturbed beetles rupture the gland by rubbing with hind tarsus, leading to the release of the secretion.

In the larvae, defensive glands consist of a pair of pouches placed in a conical depression on the second and third sternites (Figure 1b). Both pair of glands are evaginations of the sternal membrane of the second and third sternites. Each gland opens to the exterior dorsolaterally through a long and narrow channel. Glands are non-eversible and upon disturbance the secretion is discharged without eversion. The secretion is produced by gland cells that are dispersed over the entire dorsal surface of the reservoirs. Glands occur in parallel rows along the segment and at right angles to the long axis of the body. The glands in larvae are visible externally as a pair of conical lateral swellings on either side of midline in second and third abdominal segments (Figure 2b). These paired lateral swellings correspond to the structures described as of unknown function by Hayashi (1964).

Classifying the adult tenebrionid defensive glands, Tschinkel and Doyen (1980) indicate that *Luprops* deviates from the typical Lagriine type. The present study shows that the gland is more of Tenebrio type with a pair of conical reservoirs, opening to a common area of discharge and are devoid of exit ducts.
